# Supplementary material for: Sugar alcohols have the potential as bee‐safe baits for the common wasp
Source: Pest Manag Sci. 2022 May 5;78(7):3005–11. doi: 10.1002/ps.6925 (PMC9324110; doi:10.1002/ps.6925)
Supplement: Supplementary file 1 — Figure S1. Water only‐fed wasps do not prefer one side over the other and only few individuals survive. Table S1. Preference indices and survival rates. Related to Figures 2 and 3. [file PS-78-3005-s001.docx]

SUPPORTING INFORMATION

|  |
| --- |
|  |

| 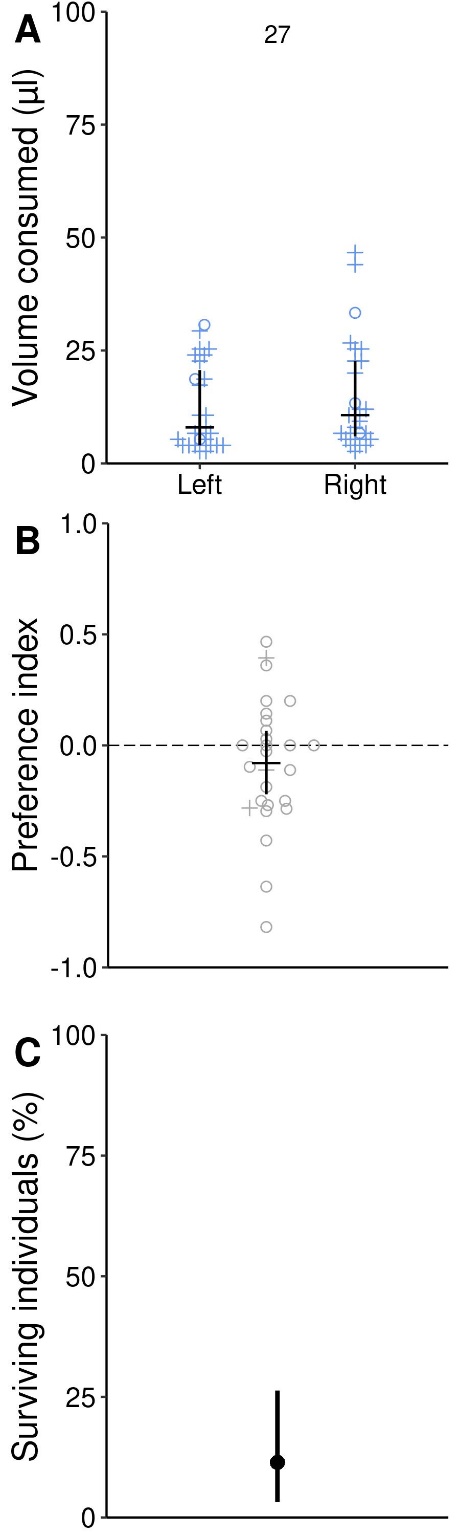 | **Figure S1. Water only-fed wasps do not prefer one side over the other and only few individuals survive.** **A**) Water consumption in a two-choice capillary feeder assay where both, the left and right capillary contained water. Symbols indicate if an individual was alive (circles) or dead (crosses) after 24h. Horizontal lines indicate the median, and vertical lines indicate 25% and 75% quantiles. Number above datapoints indicate number of animals (each animal is represented by two data points, one for each capillary). **B**) Preference index for the data in A. Positive preference indices represent a preference for water on the left side, and negative preference indices represent a preference for water on the right side. The horizontal black line indicates the estimated average of the preference index and the vertical line indicates 95% credible interval. The preference index was -0.08 (credible interval: -0.22 to 0.07) indicating that wasps had no preference for one of the sides. **C**) Survival after 24h was low (three out of 27 individuals survived). Dot indicates estimated average of preference index and vertical line indicates 95% credible interval. |
| --- | --- |

| **Table S1. Preference indices and survival rates.** Related to Figures 2 and 3. |
| --- |
|  |
